# Betaine Supplementation Improves the Production Performance, Rumen Fermentation, and Antioxidant Profile of Dairy Cows in Heat Stress

**DOI:** 10.3390/ani10040634

**Published:** 2020-04-07

**Authors:** Ali Mujtaba Shah, Jian Ma, Zhisheng Wang, Huawei Zou, Rui Hu, Quanhui Peng

**Affiliations:** 1 “Low Carbon Breeding Cattle and Safety Production” University Key Laboratory of Sichuan Province, Animal Nutrition Institute, Sichuan Agricultural University, Chengdu 61130, China; alimujtabashah@sbbuvas.edu.pk (A.M.S.); Crazyma0411@163.com (J.M.); zhwbabarla@126.com (H.Z.); ruitianhu@yeah.net (R.H.); pengquanhui@126.com (Q.P.); 2 Department of Livestock Production, Shaheed Benazir Bhutto University of Veterinary and Animal Sciences, Sakrand 67210, Sindh, Pakistan

**Keywords:** Holstein cows, summer season, rumen fermentation, feed additive

## Abstract

**Simple Summary:**

Heat stress affects the production performance of dairy cows in the summer season. Through environmental modification methods, the production performance of dairy cows can be increased, but the cost of these techniques is high, and poor farmers cannot use such techniques because of the high price. The use of feed additive may be a cost-effective and easily accessible way to decrease heat stress in animals, and it can increase the production performance of dairy cows. The use of betaine as a supplement increases milk production, rumen fermentation, and apparent digestibility of dairy cows in heat stress.

**Abstract:**

The aim of the current research was to investigate the effects of betaine (Bet) supplementation on the production performance, rumen fermentation, digestibility, and serum indexes of dairy cows. Thirty healthy Holstein cows with the same parity (milk production = 22 ± 2.5 kg) were randomly selected and divided into three groups. One group served as a control group (CON; no betaine); the other two groups were Bet1 (15 g/d per cow) and Bet2 (30 g/d per cow). All cows were fed regularly three times a day at 06:00, 14:00, and 22:00 h. Cows received the formulate diet, and water was provided ad libitum. The experiment lasted for 60 days during the summer season. Results showed that the dry matter intake, milk protein, and fat of Bet1 cows was significantly higher (*p* < 0.05) than that in other groups. The content of volatile fatty acid (VFA) in Bet1 was significantly higher (*p* < 0.05) than CON. Consistent with VFA, a similar trend was found in acetate, while propionate exhibited an opposite trend. Compared to other groups, the microbial protein (MCP) concentrations of Bet1 increased (*p* < 0.05). The apparent digestibility of dry matter (DM), organic matter (OM), crude protein (CP), neutral detergent fiber (NDF), and acid detergent fiber (ADF) of Bet1 was significantly higher (*p* < 0.05) than CON. The serum concentration of total antioxygenic capacity (T-AOC) in Bet1 and Bet2 was significantly increased (*p* < 0.05). Furthermore, the contents of malonaldehyde (MDA) and superoxide dismutase (SOD) in Bet2 were higher (*p* < 0.05) than that in other groups. Compared to CON and Bet2, Bet1 significantly increased (*p* < 0.05) the serum concentrations of glucose. Therefore, it is practicable to feed betaine to lactating cows to improve their performance in heat stress.

## 1. Introduction

Dairy cows reach optimum production performance in temperatures from 5 to 25 °C. In high environmental temperature and humidity, dairy cows cannot dissipate body heat to prevent a rise in body temperature [1,2]. Generally, dairy cow production performance is affected by a diminished feed intake when the temperature–humidity index (THI) exceeds 72 [3]. Environmental conditions and lactation are the main physiological stressors for dairy cows in the summer season and influence the normal biological functions of the animals [4]. Dairy cows dissipate body heat through their core surfaces and transfer the heat to the environment [5]. Through the environmental modification method, the production performance of dairy cows can be increased, but the cost of this technique is high [6]. The use of feed additive may be a cost-effective and easily accessible method to reduce heat stress in animals, and it can improve the production performance of dairy cows. 

Betaine (trimethylglycine) has numerous functions that may decrease the effect of heat stress (HS) in lactating dairy cows and increase milk production. For example, betaine (Bet) is an osmolyte [7] and a methyl donor [8]. It performs as a molecular chaperone [1], reduces the vulnerability of microbes to stress [9], and under some conditions, has antimicrobial activity [1]. In addition, Bet is an amino acid and has the ability to improve the production performance of different animals [1], such as steers [10] and pigs [11]. These results suggest that betaine has the potential to reduce heat stress by reducing energy expenditure [6], thereby reducing metabolic heat production and maintaining osmotic balance in animals facing heat stress.

Heat stress potentially affects the health as well as the production performance of dairy cows. Therefore, most available studies have focused on overcoming this limitation. The present research was designed to investigate the impact of betaine additive on the production performance, rumen fermentation, and antioxidant profile of dairy cows.

## 2. Materials and Methods

### 2.1. Animal Ethical Statement 

Animal research was conducted according to the rules and regulations of laboratory of animals (2017 revision) circulated by the state council (Decree No. 676). All techniques concerning animal care and management were in harmony with and permitted (Code-SYXK-Chuan-2014-184) by Sichuan Agricultural University, Chengdu, Sichuan, China.

### 2.2. Experimental Design and Diets 

The present study was conducted at a commercial dairy farm in Sichuan Province, China, with approximately 1000 milking cows from July to September 2018. A total of 30 Holstein dairy cows (bodyweight: 550 ± 18 kg) with similar daily milk production (22 ± 2.5 kg) and the same parity (second) were used and separated from the herd in this study. The selected cows were marked with ear tags and randomly allocated into three dietary treatment groups, each with 10 replicates: control (CON; no Bet), Bet1 15 g/d per cow, and Bet2 30 g/d per cow (Shanxi Xinliyuan Biotechnology Co. Ltd., Taiyuan, China).

Each cow was fed regularly three times daily at 06:00, 14:00, and 22:00 h with a total mixed ration (TMR). Average daily feed intake was measured as the difference between the amount of feed provided and rejected. All cows were mechanically milked (Afimilk system, Kibbutz Afikim, Shvil, 1514800, Israel) three times daily at 04:00, 12:00, and 20:00 h. The diet and water were provided to cows ad libitum. Before the start of the trial, a 10-day adaptation period was provided to the cows. The duration of the trial was 60 days. Betaine was supplemented in the concentrate and mixed with other ingredients. The basal diet was formulated according to National research council (NRC 2001) [12], and the ratio of concentrate to roughage was 55:45. The compositions of TMR and nutrient level are presented in Table 1.

### 2.3. Measurement of Temperature–Humidity Index (THI)

Wet-bulb and dry-bulb thermometers were hung in the barn at a distance of 1.5 m off the ground. Every day at 06:00, 08:00, 10:00, 12:00, 15:00, 17:00, and 19:00 h, temperature and humidity data were recorded. The formula THI = 0. 72 × (Td + Tw) + 40.6 was used, where Td represents dry-bulb temperature and T_W_ represents wet-bulb temperature [13]. The average of the two thermometer readings was recorded. During the whole test period, the average THI of the barn was over 72, and the highest THI was up to 88.7, indicating that the cows were in a state of heat stress during the experiment.

### 2.4. Sample Collection

The amount of feed provided and the orts were measured every day. Dry matter intake (DMI) was calculated based on the DM content in feed provided and daily ort measurements. The cows were fed individually, and the DMI of each cow was measured as feed offered and refused to calculate the average daily DMI per cow [14]. Over the last 3 d of the experiment, fecal samples (about 300 g) were obtained by stimulating the rectum to cause emissions at 02:00, 08:00, 14:00, and 20:00 h on day 58; at 00:00, 06:00, 12:00, and 18:00 h on day 59; and at 22:00, 04:00, 10:00, and 16:00 h on day 60 [15]. Meanwhile, feed and orts were sampled daily. Daily fecal samples, feed, and orts were composited per cow, subsampled, and stored at −20 °C until analysis. At the end of the trial, all samples were thawed (100 g fecal samples were mixed with 10 mL of 10% sulphuric acid) and dried for 48 h at 65 °C to a constant weight. The dried samples were smashed to pass through a 1 mm sieve (Aizela Electric Appliance Co. LTD., Zhejiang, China) for later analysis.

The daily milk yield was measured, and 40 mL milk samples were obtained three times throughout the day and mixed in the proportion of 4:3:3 in the morning, afternoon and evening on day 60. The composition of fat, lactose, protein, urea nitrogen, and somatic cell count was examined immediately by an automatic multifunctional dairy analyzer (Botong Ruihua Scientific Instrument Co. LTD, Beijing, China). Blood was collected from all cows prior to morning feeding on day 60 using evacuated tubes containing no anticoagulant, and blood samples were obtained from the caudal vein and centrifuged for 15 min at 3500 × g (4 °C) to harvest serum. All serum samples were collected in 1.5 mL microtubes and stored at −20 °C until analysis.

Rumen fluid samples were obtained by a flexible esophageal tube (Anscitech Co. Ltd., Wuhan, China) from all cows 4 h after the morning milk feeding [16] on day 60. Ruminal fluid was strained by four layers of cheesecloth, and the pH of rumen was measured immediately with a portable pH meter (PH200, Ruizhen Electronic Technology Co., Ltd., Shanghai, China). After measurement of pH, 10 mL of strained ruminal fluid was shifted into sterile tubes containing 1 mL of 25% metaphosphoric acid. This solution was gently hand-shaken and kept at −20 °C for later analysis.

### 2.5. Chemical Analysis and Calculations

The ground samples (diets, orts, and feces) were analyzed for DM (method 930.15), ether extract (EE) (method 920.39), organic matter (OM) (method 942.05), and crude protein (CP) (method 984.13) according to the procedures of Association of Official Analytical Chemists (AOAC 1998) [17]. The neutral detergent fiber (NDF) and acid detergent fiber (ADF) contents were determined according to a method described by Van Soest et al. [18] using a heat-stable amylase. The apparent total tract digestibility (D, %) of dietary nutrients was measured using the acid-insoluble ash (AIA) ratio technique. The AIA in feces (Af, %) and diets (Ad, %) were analyzed using the method described by Van Keulen and Young [19]. With the content of a nutrient in feces (Nf, %) and diet (Nd, %), the apparent nutrient digestibility was determined using the equation as follows: D = (1 − (Ad × Nf)/(Af × Nd)) × 100.

Blood serum samples were analyzed for blood biochemical indexes, including non-esterified fatty acid (NEFA), β-hydroxybutyric acid (BHBA), and glucose (GLU), using commercial kits (Jiancheng Bioengineering Institute, Nanjing, China) and an automatic biochemical analyzer (SHIMADZU, Kyoto, Japan).

The serum antioxidant indices including total antioxygenic capacity (T-AOC), glutathione peroxidase (GSH-Px), malonaldehyde (MDA), and superoxide dismutase (SOD) were determined by using commercial kits (Jiancheng Bioengineering Institute, Nanjing, China) and a spectrofluorophotometer (Shimadzu, Kyoto, Japan). 

Frozen rumen fluid samples were thawed and then centrifuged at 15,000 × g for 10 min at 4 °C, and the supernatants were analyzed for volatile fatty acid (VFA) by gas chromatography (Agilent Technologies, Santa Clara, CA, USA) [20], microbial protein (MCP) by the method of Makkar et al. [21], and ammonia N concentrations by alkaline sodium hypochlorite-phenol spectrophotometry [22].

### 2.6. Statistical Analysis 

One-way ANOVA (SPSS statistical software, Ver. 20.0 for Windows; SPSS, Chicago, IL, USA) was used to analyze the data. The statistically significant differences were determined by Duncan’s multiple range test. Data were presented as mean and SEM. The significance level was indicated at *p* < 0.05, and trend was declared at 0.05 ≤ *p* < 0.10.

## 3. Results

### 3.1. Lactation Performance

The DMI, milk yield, and milk fat of Bet1 were significantly higher (*p* < 0.05) than those of other groups (Table 2); however, lactose was similar. Compared to other groups, the somatic cell count (SCC) of CON was more than 300 × 103/mL and significantly higher (*p* < 0.05) than that of other groups.

### 3.2. Rumen Fermentation

The results of rumen fermentation are mentioned in Table 3. The ruminal pH of all groups was less than 7, and they had numerical differences. The content of VFA in Bet1 was higher and significant (*p* < 0.05) than that of CON. Consistent with VFA, a similar trend was found in acetate, while propionate exhibited a significant difference (*p* < 0.05). The content of butyrate was significantly (*p* < 0.05) higher and the ratio of acetate to propionate was significantly different (*p* < 0.05) in the Bet-supplemented group as compared to those of CON. However, the content of ammonia N in CON was significantly higher (*p* < 0.05) than that of other groups. Compared to other groups, the MCP concentrations of Bet1 increased (*p* < 0.05) dramatically. 

### 3.3. Nutrient Apparent Digestibility

Data for nutrient apparent digestibility are displayed in Table 4. The apparent digestibility of DM, OM, CP, NDF, and ADF in Bet1 was significantly higher (*P*<0.05) than that of CON. No noticeable difference was found in EE apparent digestibility among all groups.

### 3.4. Serum Parameters

**Figure 1:**
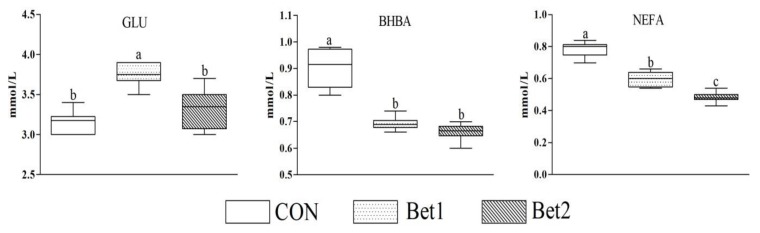
Effects of Bet on the serum GLU, NEFA, and BHBA parameters of dairy cows. GLU = glucose; NEFA = non-esterified fatty acid; BHBA = β-hydroxybutyric acid.

The results of the antioxidant capacity of dairy cows are mentioned in Table 5. The serum concentration of T-AOC in Bet1 and Bet2 was significantly increased (*p* < 0.05). Furthermore, the contents of MDA and SOD in Bet2 were greater (*p* < 0.05) compared to other groups. However, compared to CON and Bet2, the GSH-Px concentrations of Bet1 were highest (*p* < 0.05). The serum concentrations of GLU, BHBA, and NEFA are shown in Figure 1. Compared to CON and Bet2, Bet1 had significantly increased (*p* < 0.05) serum concentrations of GLU. Cows fed Bet had reduced serum BHBA and NEFA concentrations.

## 4. Discussion 

In a high environmental temperature, dairy cows cannot maintain their body temperature through heat release, resulting in an upsurge in rectal temperature. Gaughan et al. [23] suggested that the supplementation of exogenous substances significantly decreases rectal temperature and improves the intake of feed and heat endurance of cows in summer heat stress.

In the present study, DMI, milk yield, and composition of the milk increased with supplementation of 15 g of betaine per day in heat stress conditions. Cows received 15 g of betaine supplementation per day consumed more TMR than that of the control group. Higher environmental temperature and THI decreased the milk production performance of dairy cows with a log of 1 to 3 days after THI [24]. Hall et al. [25] stated that the addition of up to 150 g of betaine per day in TMR increases milk production in a linear manner in thermoneutral conditions. Nevertheless, the impact of betaine supplementation on lactating dairy cows in the hot season, or during the summer season, are more equivocal [2], possibly due to the multifaceted dose-dependent responses to betaine [20]. For example, milk responses to betaine supplementation during heat stress were highest at 15 g betaine per day and, indeed, disappeared at doses above this [2]. Consistent with these results, Hall et al. [25] stated that supplementation of betaine at 35 and 70 g/d improved milk production in thermoneutral conditions but not in heat stress. Curvilinear dose responses to betaine supplementation have also been noticed in beef cattle and sheep during HS with improvements at 2 and 15 g betaine per day, respectively [6,10]. The reason for the moderation in response to high doses of Bet during HS may be that the stimulation of hepatic metabolism and a resultant upsurge in heat production by the liver may offset the decrease in heat production due to the osmoprotective effects of betaine in the rest of the body [1].

VFA, acetate, and propionate concentrations increased with the supplementation of betaine in the present study, so it is possible that betaine supplementation could increase rumen fermentation by serving as a source of either ruminally available nitrogen or methyl groups [26]. The results of the present study are in line with previous research by Dunshea et al. [1]. Wdowiak-Wróbel et al. [27] reported that betaine supplementation has an osmoprotective effect, and it promotes the growth of favorable microbiota in rumen under environmental stress conditions. It is also noticed that rumen pH fluctuates with betaine supplementation, and this may extend to heat stress conditions, subsequently increasing the temperature of rumen in HS [24]. Peterson et al. [28] demonstrated that betaine is metabolized in rumen and converted into acetate, which may play an essential role in fat synthesis. The results from the present study show that betaine supplementation improved rumen fermentation.

Supplementation of betaine increased the apparent digestibility of DM, OM, CP, NDF, and ADF, and no effect was observed in EE among the groups during the heat stress condition in dairy cows. The findings of the present study are in line with the previous study of Wang et al. [29] who described that dietary betaine improved the digestibility of the nutrients. This may be due to the fact that betaine is an osmolyte and methyl donor, which decreases heat stress, maintains the pH of the rumen, and also improves the ruminal microbial community [30]. 

In the present study, glucose levels in serum increased with the supplementation of betaine; this may be due to increased feed intake and digestibility in the gastrointestinal tract. Serum NEFA levels can serve as energy balance and energy utilization indicators in cows [29]. In the current study, the linearly decreased NEFA levels in plasma suggest that the availability of energy improved with the supplementation of betaine. However, Davidson et al. [31] reported that supplementation with 45 g rumen-protected betaine per day with corn silage-based TMR (TMR was limited in methionine) to lactating Holstein cows from 21 to 91 days in milk (DIM) did not affect plasma NEFA and BHBA contents. 

The formation of endogenous free radicals increases in dairy cows in heat stress conditions and decreases the animal’s antioxidant capacity. These variations are echoed in serum by the action of antioxidant enzymes, with a significant decrease in SOD and GSH-Px activities and an upsurge in concentrations of reactive oxygen species (ROS) and MDA. Earlier studies found that the antioxidant capacity of betaine enabled it to scavenge free radicals and protect cells from loss in rats [32]. In the present research, the supplementation of betaine at 15g per day improved the GSH-Px levels in blood plasma. In contrast, MDA and SOD levels were augmented by the addition of 30 g per day of betaine. These findings show a decrease in ROS and free radicals along with an improvement in antioxidant capacity. With an increase in betaine, ROS and free radicals increased, thereby increasing SOD and MDA levels. Therefore, 15 g/d was the optimized dosage.

## 5. Conclusions 

Supplementation of betaine increased the milk yield, rumen fermentation, and apparent digestibility of dairy cows in heat stress. In addition, betaine supplementation improved the antioxidant profile and serum metabolites in dairy cows. In this research, we conclude that 15 g of betaine per day can lead to better production performance of dairy cows in heat stress conditions.

## Figures and Tables

**Table 1:** Basal diet composition and nutrient levels (DM basis, %).

Ingredient	Contents	Item	Contents
Ingredients		Nutrient Levels	
Corn silage	36.80	NEg (MJ/kg) ^2^	5.48
Alfalfa hay	3.45	CP	14.88
Chinese wildrye	6.01	NDF	43.15
Corn	16.67	ADF	25.20
Soybean meal	3.85	Ca	0.89
Beet pulp	4.28	P	0.58
Brewer’s grains	16.55		
DDGS	3.44		
Cottonseed meal	3.48		
Molasses	3.66		
Limestone	0.35		
CaHPO_4_	0.40		
Na_2_CO_3_	0.53		
NaCl	0.33		
Premix ^1^	0.20		
Total	100		

DDGS = distillers dried grains with soluble; CP = crude protein; NDF = neutral detergent fiber; ADF = acid detergent fiber. ^1^ The premix provided the following per kg of the diet: VA 7500 IU, VD 1300 IU, VE 50 IU, Cu (as copper sulfate) 10 mg, Fe (as ferrous sulfate) 100 mg, Mn (as manganese sulfate) 40 mg, Zn (as zinc sulfate) 60 mg, I (as potassium iodide) 0.50 mg, Se (as sodium selenite) 0.3 mg, and Co (as cobalt chloride) 0.1 mg. ^2^ NEg was calculated according to the Nutrient Requirements of Dairy Cattle: Seventh Revised Edition, 2001. ^1^ The premix provides per kg diet.

**Table 2:** Effect of betaine on lactation performance of dairy cows.

Item	CON	Bet1	Bet2	SEM	*p*
DMI (kg/d)	18.83	19.99	18.90	0.158	0.081
Milk yield (kg/d)	21.98	23.24	22.77	0.114	<0.001
Milk fat (%)	4.02	4.11	4.03	0.016	0.036
Milk protein (%)	3.12	3.15	3.13	0.006	0.041
Lactose (%)	4.54	4.57	4.56	0.011	0.562
SCC (×103/mL)	392.20	289.80	297.20^b^	7.808	<0.001

Bet1 = betaine 15 g/d per cow; Bet2 = betaine 30 g/d per cow; DMI = dry matter intake; SCC = somatic cell count. In the same row, values with different letters mean significant difference (*p* < 0.05).

**Table 3:** Effect of betaine on rumen fermentation of dairy cows.

Item	CON	Bet1	Bet2	SEM	*p*
pH	6.55	6.54	6.42	0.028	0.346
VFA (mmol/L)	117.20	127.00	122.40	2.208	0.034
Acetate (mmol/L)	68.40	71.00	72.10	0.386	<0.001
Propionate (mmol/L)	22.90	20.60	20.80	0.298	0.003
Butyrate (mmol/L)	11.69	15.33	13.47	0.282	<0.001
Acetate-to-propionate ratio	3.00	3.46	3.48	0.057	0.012
NH_3_-N (mg/dL)	15.44	11.20	10.77	0.395	<0.001
MCP (mg/mL)	6.55	8.69	7.21	0.469	<0.001

Bet1= betaine 15 g/d per cow; Bet2 = betaine 30 g/d per cow; MCP = microbial protein; VFA = volatile fatty acid. In the same row, values with different letters mean significant difference (*p* < 0.05).

**Table 4:** Effect of betaine on the apparent digestibility of dairy cows.

Item	CON	Bet1	Bet2	SEM	*p*
DM (%)	72.44	75.00	73.40	0.213	<0.001
OM (%)	62.15	65.30	64.21	0.948	0.035
CP (%)	65.82	72.25	69.55	0.498	<0.001
EE (%)	71.04	70.14	70.05	1.072	0.358
NDF (%)	51.65	54.41	53.84	0.866	0.033
ADF (%)	44.50	48.24	45.28	0.303	<0.001

Bet1 = betaine 15 g/d per cow; Bet2 = betaine 30 g/d per cow; DM = dry matter; OM = organic matter; CP = crude protein; EE = ether extract; NDF = neutral detergent fiber; ADF = acid detergent fiber.

**Table 5:** Effect of betaine on the serum antioxidant parameters of dairy cows.

Item	CON	Bet1	Bet2	SEM	*p*
T-AOC (U/L)	1.06	2.32	2.39	0.613	<0.001
GSH-Px (U/L)	116.06	153.74	93.24	4.64	<0.001
MDA (U/L)	1.26	2.09	2.39	0.089	<0.001
SOD (U/L)	13.55	14.40	16.43	0.235	<0.001

Bet1 = betaine 15 g/d per cow; Bet2 = betaine 30 g/d per cow; T-AOC = total antioxygenic capacity; GSH-Px = glutathione peroxidase; MDA = malonaldehyde; SOD = superoxide dismutase; U/L = units per liter.
